# Associations between cigarette smoking and cannabis dependence: A longitudinal study of young cannabis users in the United Kingdom

**DOI:** 10.1016/j.drugalcdep.2015.01.004

**Published:** 2015-03-01

**Authors:** Chandni Hindocha, Natacha D.C. Shaban, Tom P. Freeman, Ravi K. Das, Grace Gale, Grainne Schafer, Caroline J. Falconer, Celia J.A. Morgan, H. Valerie Curran

**Affiliations:** a Clinical Psychopharmacology Unit, University College London, Gower Street, London WC1E 6BT, United Kingdom; b Department of Psychology, University of Exeter, Washington Singer Building, Perry Road, Exeter EX4 4QG, United Kingdom; c Department of Clinical, Educational and Health Psychology, University College London, Gower Street, London WC1E 6BT, United Kingdom

**Keywords:** Cannabis, Tobacco, Addiction, United Kingdom, Longitudinal, Dependence, Co-morbidity

## Abstract

**Aims:**

To determine the degree to which cigarette smoking predicts levels of cannabis dependence above and beyond cannabis use itself, concurrently and in an exploratory four-year follow-up, and to investigate whether cigarette smoking mediates the relationship between cannabis use and cannabis dependence.

**Methods:**

The study was cross sectional with an exploratory follow-up in the participants’ own homes or via telephone interviews in the United Kingdom. Participants were 298 cannabis and tobacco users aged between 16 and 23; follow-up consisted of 65 cannabis and tobacco users. The primary outcome variable was cannabis dependence as measured by the Severity of Dependence Scale (SDS). Cannabis and tobacco smoking were assessed through a self-reported drug history.

**Results:**

Regression analyses at baseline showed cigarette smoking (frequency of cigarette smoking: *B* = 0.029, 95% CI = 0.01, 0.05; years of cigarette smoking: *B* = 0.159, 95% CI = 0.05, 0.27) accounted for 29% of the variance in cannabis dependence when controlling for frequency of cannabis use. At follow-up, only baseline cannabis dependence predicted follow-up cannabis dependence (*B* = 0.274, 95% CI = 0.05, 0.53). At baseline, cigarette smoking mediated the relationship between frequency of cannabis use and dependence (*B* = 0.0168, 95% CI = 0.008, 0.288) even when controlling for possible confounding variables (*B* = 0.0153, 95% CI = 0.007, 0.027).

**Conclusions:**

Cigarette smoking is related to concurrent cannabis dependence independently of cannabis use frequency. Cigarette smoking also mediates the relationship between cannabis use and cannabis dependence suggesting tobacco is a partial driver of cannabis dependence in young people who use cannabis and tobacco.

## Highlights

• We studied the extent that cigarette smoking predicts level of cannabis addiction.
• We tested whether cigarette smoking mediates the effect of cannabis use on dependence.
• We interviewed 298 cannabis and tobacco users, of which 65 were followed up.
• Cigarette smoking accounted for 29% of the variance in cannabis dependence.
• Cigarette smoking mediated the relationship between cannabis use and dependence.

## 1 Introduction

Together, cannabis and tobacco are two of the world's most used drugs, and despite their unique smoking relationship, relatively little is known about their combined effects. The high prevalence of cannabis use amongst young people in the UK is a growing concern. However, many daily cannabis users do not develop dependence. Prospective studies of the likelihood of developing a Cannabis Use Disorder (CUD) have investigated predictors of dependence amongst cannabis users (Swift et al., 2000; van der Pol et al., 2013) with baseline severity of dependence acting as a main predictor of dependence at one-year follow-up (Swift et al., 2000). However, there are a host of other factors which have been considered predictors of developing a CUD, for example; age of onset (Chen et al., 2005), gender (Coffey et al., 2000; von Sydow et al., 2002), impulsivity (Swift et al., 2008), mental health problems (Wittchen et al., 2007) and early onset of continued tobacco smoking (Coffey et al., 2000; Prince van Leeuwen et al., 2014; von Sydow et al., 2002). More recently, van der Pol et al. (2013) investigated a population of high risk young adult cannabis users and found that recent negative life events and social support factors such as living alone were more predictive of CUD then cannabis exposure variables suggesting the existing literature on the aetiology of cannabis use disorder is limited.

Relatively, tobacco is more harmful than cannabis (Nutt et al., 2010) and the majority of tobacco smokers are indeed nicotine dependent. The gateway hypothesis posits that tobacco acts as a gateway drug to the use of cannabis (Kandel et al., 1992). However, there is strong evidence for the ‘reverse gateway’ whereby cannabis smoking predicts tobacco onset (Patton et al., 2005). Several lines of investigation give weight to the hypothesised association between cannabis use and tobacco smoking. Firstly, there is evidence to suggest both nicotine and cannabis affect similar mesolimbic dopaminergic pathways suggesting overlapping mechanism in addiction (David et al., 2005; Filbey et al., 2009). Secondly, there are shared genetic (Agrawal et al., 2008, 2010), temperamental (Brook et al., 2010; Creemers et al., 2009) and psychological factors (Brook et al., 2010) that have been associated with the use of both drugs. Finally, both substances are smoked and often concurrently, such that cross-sensitisation to each substance might occur, with tobacco directly enhancing the subjective effect of cannabis (Agrawal and Lynskey, 2009; Baggio et al., 2013; Ream et al., 2008). As nicotine is more addictive than cannabis, tobacco smoking may be a primary driver of continued use and relapse in co-dependent users.

About 90% of cannabis users also identify as cigarette smokers (Agrawal et al., 2012), however, this exists as a complicated relationship given that increased cigarette smoking may substitute for reduced cannabis consumption (Allsop et al., 2014) and vice versa. Users of both drugs report more severe symptoms of CUD (Peters et al., 2012). Half of adults seeking treatment for CUD also smoke cigarettes and treatment outcomes for those using both cannabis and tobacco, in comparison to cannabis alone, are poor (Agrawal et al., 2009). Moreover, relative to those with a CUD, those with co-occurring nicotine dependence show poorer psychiatric and psychosocial outcomes (Peters et al., 2014; Ramo et al., 2013). In a recent controlled laboratory study, Haney et al. (2012) found that the strongest predictor of relapse in cannabis dependent individuals was their cigarette smoking status. Further, cigarette smoking ad libitum or after a short period of abstinence were both associated with relapse to cannabis use thus ruling out acute nicotine exposure or conditioned motivation (i.e., transfer) effects. This study suggests that cigarette smoking alongside cannabis use may confer a greater dependence syndrome and therefore a greater likelihood to relapse.

To understand the factors involved in the maintenance of substance use, such that prevention strategies are better informed, longitudinal designs of the use of both drugs are essential, especially during the critical period of adolescence. The present study aimed to investigate the degree to which cigarette smoking predicts the level of cannabis dependence above and beyond cannabis use itself, both at baseline, and in an exploratory four-year follow-up in a sample of young cannabis and tobacco users. Cigarette smoking at baseline, independently of smoking cannabis, is hypothesised to contribute to CUD concurrently and at follow up. Moreover, following previous research (Haney et al., 2012) we aimed to investigate if the effects of cannabis use on cannabis dependence are mediated by tobacco smoking using a multiple mediator model.

## 2 Methods

### 2.1 Design and participants

#### 2.1.1 Baseline

A sample of 298 cannabis users who also used tobacco (≥1 day/month) were selected from a sample comprising of over 400 recreational (1–24 days/month) and daily (≥25 days/month) users aged 16–23 years old, as described elsewhere (Freeman et al., 2014; Morgan et al., 2012). Inclusion criteria were (a) to speak English fluently, (b) not to have learning impairments, (c) to have no history of psychotic illnesses and (d) normal or corrected-to-normal vision. All participants provided written, informed consent. Participants could also consent to be contacted for further studies and provided contact details as such. The study was approved by the UCL Ethics Committee and its aims were supported by the UK Home Office.

#### 2.1.2 Procedure

Baseline measures were collected in participants’ homes as part of a larger study investigating acute cannabis effects. Participants were required to abstain from all recreational drugs including alcohol for 24 h before each test day. Demographic information, a drug history and assessment of CUD, via the Severity of Dependence Scale (SDS; Gossop et al., 1995), were completed while participants were abstinent. Participants’ past use of cannabis and tobacco were assessed using a semi-structured, questionnaire-based interview which included the following questions: (a) when did you last use tobacco? (b) For how many years have you smoked tobacco? (YEARS-TOB) (c) In a typical month, how many days do you use tobacco? (DAYS-TOB) (d) How many cigarettes do you smoke per day? (e) When did you last use cannabis? (f) For how many years have you used cannabis? (g) In a typical month, how many days do you use cannabis? (DAYS-CANNABIS) (h) How long does it take you to smoke an eighth (3.5 g)?

Participants were assessed for cannabis dependence using the SDS which is five-item questionnaire focusing on ‘loss of control’ or ‘psychological dependence’ in relation to cannabis use. It has good and well-established psychometric properties and was found to be of equal utility in diagnosing cannabis dependence in comparison to more formal diagnostic assessments (Swift et al., 1998). A score of three on the SDS indicates cannabis dependence (Swift et al., 1998). The following measures were also administered; (a) the Wechsler Test of Adult Reading (WTAR; Wechsler, 2001) which is a measure of premorbid verbal intelligence (IQ) and consists of 50 irregularly spelt words. Scores range from 0 to 50; (b) the Schizotypal Personality Questionnaire (SPQ; Raine, 1991) which is a 74-item questionnaire where higher scores indicate a greater schizotypal personality disorder severity; (c) the State-Trait Anxiety Inventory (STAI; Spielberger, 1983), only the 20 items from the trait scale were administered with higher scores reflecting greater trait anxiety; (d) the Barratt Impulsiveness Scale (BIS-11; Patton et al., 1995) which is a 30 item questionnaire describing common impulsive behaviours, high scores reflect greater impulsivity; (e) the Beck Depression Inventory (BDI; Beck et al., 1961) which is a 21 item questionnaire indexing depression over the past week (a score of 10 indicates mild depression) and (f) the Childhood Trauma Questionnaire (CTQ; Bernstein et al., 2003) which is a 28 item questionnaire assessing history of abuse.

### 2.2 Follow up

**Fig. 1:**
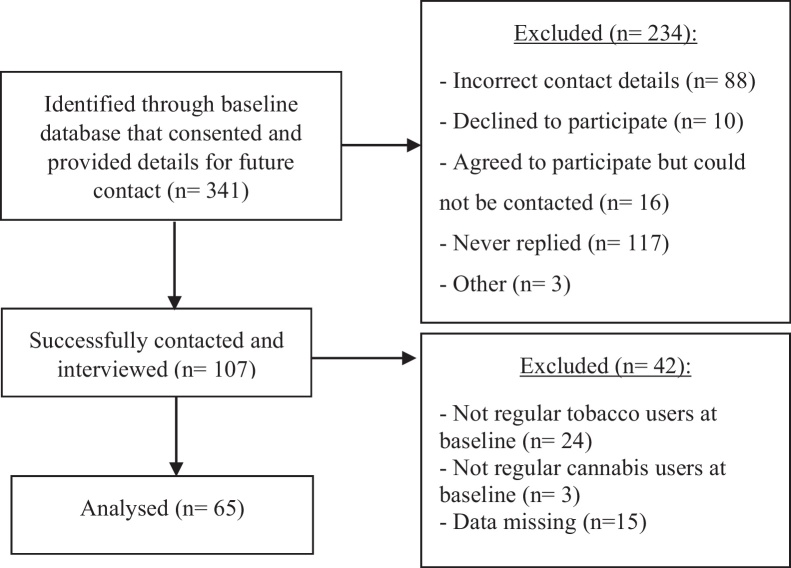
Participant flow diagram for opportunistic follow up, 4 years after baseline.

At follow-up, four years later, we attempted to re-contact the 341 participants who gave consent and invited them to participate in a semi-structured telephone interview (see Fig. 1 for participant flow diagram). The final sample consisted of 65 cannabis and tobacco smokers.

Participants were recruited through a preliminary email requesting their participation. All participants gave informed consent by telephone and were entered into a prize draw to win a tablet computer for participating. Telephone interviews were conducted between October and December 2013. Demographics, a drug history and the SDS, to reassess participants for CUD, identical to the baseline assessments, were collected.

### 2.3 Statistical analysis

All analyses were conducted in IBM Statistical Package for Social Sciences (SPSS), V.21. Assumptions of no perfect multicollinearity (no *r*s ≥ 0.8), linearity, normally distributed errors and homoscedasticity were not violated. Correlations were conducted between cannabis dependence, predictors and possible confounders. At baseline, linear regression was used to assess the predictive relationship of cannabis variables on cannabis dependence. Tobacco smoking variables were added to the regression model to establish whether they could explain significant additional variance in CUD. Questionnaire measures that correlated strongly with cannabis dependence were then added to the model and finally variables that were not found to be significant as regression coefficients were removed generating the most parsimonious model (accounting for the greatest amount of variance with the least number of variables). Those predictors were then used to predict cannabis dependence in the follow up data. Unstandardised *B* coefficients are presented with 2 decimal places.

We used PROCESS for Statistical Package for Social Sciences (SPSS) version 21 (Hayes, 2013; Preacher and Hayes, 2008). Multiple mediation analyses were conducted on a priori hypotheses. We tested the possible indirect effects of DAYS-CANNABIS on CANNABIS DEPENDENCE (SDS score) through tobacco smoking variables (YEARS-TOB + DAYS-TOB) in a multiple mediator model whist controlling for confounding variables in the baseline data. This method parses the relationship between a predictor and an outcome into ‘indirect’ and ‘direct’ effects. Indirect effects occur when the predictor influences the outcome variable through another mediator variable. Multiple mediators have a combined and a specific (individual) contribution to the relationship between a predictor and outcome. In contrast, ‘direct effects’ between the predictor and outcome are statistically independent of this mediating relationship. For all analyses we used bias corrected 95% confidence intervals (CI) which resulted from bootstrapping of 10,000 samples. An effect is deemed significant when the *B* lies within CIs that do not cross zero.

### 2.4 Missing data

For 4 participants, single questionnaire items for the SPQ (5 individual responses missing in total) were replaced with the mean of the subscale. SPQ data was missing for 11 participants. For 8 participants, single questionnaire items for the BIS (10 items in total) were replaced with the mean. For the STAI, 7 items in total were replaced with the mean. Thus, a total of 0.05% of the baseline data was replaced with mean scores.

## 3 Results

### 3.1 Baseline demographics

Participants in this study (*N* = 298; 71% male) were on average 20.55 ± 1.67 years old with 14.47 ± 1.94 total years in education. Their mean score on the BDI was 7.27 ± 6.67 with a range of 0–40 (normative values; 6.25 ± 4.00 (Crawford et al., 2011)), 44 participants (4.69%) scored >14 (mild depression), STAI 39.41 ± 9.02 (normative values; 36.44 ± 10.93 (Crawford et al., 2011)), BIS 70.73 ± 9.84 (normative values; 64.2 ± 10.70 (Spinella, 2007)), WTAR 41.93 ± 6.80, CTQ 37.09 ± 10.04 (normative values; 98.63 ± 29.13 (Paivio and Cramer, 2004)) SPQ (*N* = 287) 17.67 ± 10.70 (normative values; 26.9 ± 11.00 (Raine, 1991)). 139 (46.6%) participants met criteria for cannabis dependence (score ≥3 on the SDS) at baseline.

### 3.2 Follow up demographics

The follow up sample (*N* = 65; 69.2% male) were a mean (SD) age of 24.66 ± 2.07. 26.2% (*N* = 17) met the criteria for cannabis dependence at follow-up (further details of baseline demographics for this group can be found in Table 3). In comparison to the 233 who were not followed up, the 65 who were did not differ significantly on age, gender, primary study variables or smoking characteristics suggesting that the baseline demographics of the follow up group are equivalent to the baseline group who were not followed up.

### 3.3 Correlations of primary baseline variables (Table 1)

Correlations were conducted between the outcome variable of SDS score (cannabis dependence), predictors and possible confounders (Table 1). SDS correlated positively with scores on the BDI (*r* = 0.690, *p* = 0.003). SDS correlated weakly with the WTAR (*r* = −0.16, *p* = 0.004) and also weakly with scores on the SPQ (*r* = 0.133, *p* = 0.024) but not on the STAI (*r* = 0.090, *p* = 0.133) or BIS (*r* = 0.090, *p* = 0.110). BIS scores correlated with cigarettes per day (*r* = 0.166, *p* = 0.004) and days per month cannabis use (*r* = 0.153, *p* = 0.008).

### 3.4 Regression analysis (Table 2)

#### 3.4.1 Cannabis only model

This model predicted 24.6% of the variance in cannabis dependence. Cannabis dependence score was significantly predicted by DAYS-CANNABIS. Cannabis dependence scores increased by 0.12 units for every extra day of cannabis use per month. Time to smoke an eighth, years of cannabis use and days since last cannabis use were not predictive of cannabis dependence.

#### 3.4.2 Cannabis + tobacco model

When tobacco variables are added to regression model, the model predicted 28.5% of the variance in cannabis dependence (*R*^*2*^ *change* = 0.038, *F change*(4,298) = 3.880, *p* = 0.004). DAYS-CANNABIS remained a significant predictor of cannabis dependence with dependence scores increasing 0.1 units for every extra day per month. YEARS-TOB was predictive of cannabis dependence. For every additional year of tobacco smoking, cannabis dependence scores increased 0.197 units. DAYS-TOB was a significant predictor of cannabis dependence; scores increased by 0.031 units for every additional day of tobacco use per month. Time to smoke an eighth, years cannabis smoked and days since last cannabis use were not predictive of cannabis dependence.

#### 3.4.3 Cannabis, tobacco + confounders model

Variables that correlated strongly with cannabis dependence scores were added to the regression model. BDI score significantly predicted cannabis dependence. For every unit increase on the BDI, cannabis dependence scores increased by 0.046 units. As such the model predicted 30.4% of the variance in cannabis dependence scores (*R*^*2*^ *change* = 0.019, *F change*(2,287) = 3.955, *p* = 0.020).

### 3.5 Most efficient model

When redundant predictors were removed from the analysis, the model predicated 29.5% of the variance in cannabis dependence, which is not significantly different from model 3 (cannabis, tobacco + confounders) which includes cannabis, tobacco and potential confounders (*R*^*2*^ *change* = 0.090, *F change*(4,293) = 0.008, *p* = 0.750). DAYS-CANNABIS remained the most important predictor of cannabis dependence, followed by YEARS-TOB, DAYS-TOB and BDI score. In this model, *r* = 0.54 for the most efficient model which is considered a large effect size (Cohen, 1988).

### 3.6 Exploratory regression analysis

Demographic variables were added to the most efficient given the associations between these variables and CUD (as sex differences have been reported in relation to abuse related effects; Cooper and Haney, 2014). When gender is added to this model, the model predicts 29.6% of the variance in cannabis dependence (*R*^*2*^ *change* = 0.010, *F change*(1,292) = 0.180, *p* = 0.670).

Age was then added to the most efficient model (without gender). This model accounts for 30.7% of the variance in cannabis dependence (*R*^*2*^ *change* = 0.011, *F change*(1,292) = 4.740, *p* = 0.030). The addition of Age (*B* = 0.198, 95% BCI = 0.037, 0.368) correlated highly with the variable YEARS-TOB, which was no longer significant when age was added (*B* = 0.096, 95% BCI = −0.035, −0.223).

Finally, scores on the CTQ were added to the regression model. This model accounted for 28.7% in the variance of cannabis dependence (*R*^*2*^ *change* = 0.001, *F change*(1,278) = 0.440, *p* = 0.510).

### 3.7 Exploratory follow-up analysis

#### 3.7.1 Regression at follow-up (Table 4)

##### 3.7.1.1 Most efficient model

The significant predictors in the baseline regression (Table 2) were used to predict cannabis dependence at follow-up, 4 years later. This was to gage whether the same factors that predict dependence at baseline can predict dependence at follow up. Means, standard deviations and correlation coefficients of these variables can be found in Table 3. This model predicted 18.5% of the variance in dependence at follow-up. DAYS-CANNABIS, DAYS-TOB and YEARS-TOB and BDI score were not significant predictors of cannabis dependence at follow up.

#### 3.7.2 Most efficient model accounting for baseline cannabis dependence

Baseline cannabis dependence was added to the model stated above. As a result, cannabis dependence became the only significant predictor of predicted cannabis dependence at follow-up (*R*^*2*^ *change* = 0.062, *p* < 0.031). This model predicted 24.8% of the variance in dependence at follow-up.

### 3.8 Multiple mediation analysis (Fig. 2)

As a result of DAYS-TOB and YEARS-TOB being significant predictors of baseline cannabis dependence in the linear regression (Table 2), these variables were used a mediators in a multiple mediator model to discern if the relationship between cannabis use (DAYS-CANNABIS) and cannabis dependence (SDS score) was mediated by concurrent tobacco use.

**Fig. 2:**
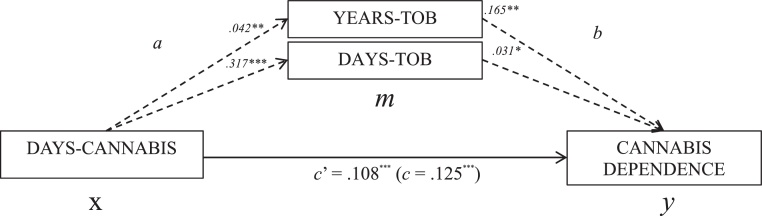
Multiple mediator model. Paths *a*, *b*, *c* and *c*′ are OLS regression coefficients in unstandardised form. Path *c*′ represents the effect of *x* on *y* when tobacco variables (*m*) are included as mediators. Path *c* represents the effect of *x* on *y* when the tobacco variables are not included as mediators. *p < .05, **p < .01, ***p < .001.

A bias-corrected and accelerated bootstrapped multiple mediation model confirmed the presence of a combined indirect effect of DAYS-CANNABIS on cannabis dependence through YEARS-TOB + DAYS-TOB (*B* = 0.017, 95% CI = 0.008, 0.288), with significant, specific indirect effects through YEARS-TOB (*B* = 0.007 95% CI = 0.002, 0.016) and DAYS-TOB (*B* = 0.010, 95% CI = 0.003, 0.020) (product of paths *a* and *b* in Fig. 2). This model accounted for 28% of the variance in cannabis dependence, whereas the direct effect of DAYS-CANNABIS on CANNABIS DEPENDENCE, accounted for 23% (direct *B* = 0.108, 95% CI = 0.008, −0.028). Pairwise comparison between specific indirect effects was not significant (*B* = −0.003, 95% CI = −0.014, 0.008) suggesting that both YEARS-TOB and DAYS-TOB are not statistically different from each other i.e. have equal importance in mediating this relationship. The direct route (c and c′) suggests that when taking into account the mediating role of tobacco smoking, DAYS-CANNABIS is still significant.

### 3.9 Influence of confounds

Given that both BDI and WTAR correlated with dependence at baseline, these were added as covariates into the above analysis. As such this model predicted 30% of the variance in cannabis dependence. The indirect effect of DAYS-CANNABIS on CANNABIS DEPENDENCE through YEARS-TOB and DAYS-TOB whilst controlling for BDI and WTAR was significant (*B* = 0.015, 95% CI = 0.007, 0.027) with specific indirect effects through YEARS-TOB (*B* = 0.007, 95% CI = 0.002, 0.015), and DAYS-TOB (*B* = 0.009, 95% CI = 0.003, 0.018), with no significant difference between DAYS-TOB and YEARS-TOB. The direct effect of DAYS-CANNABIS on CANNABIS DEPENDENCE when controlling for these covariates is still significant (*B* = 0.105, 95% CI = 0.078, 0.131).

## 4 Discussion

The main aim of this study was to investigate the role of cigarette smoking on cannabis dependence, above and beyond the effects of cannabis exposure, in a sample of young cannabis and tobacco co-users. We conducted an exploratory follow-up of these users four years later with a 27% response rate of which 70% of individuals had smoked cannabis and tobacco at baseline. The 65 participants that were followed up were equivalent in demographics and smoking behaviour to those who were not followed up, at baseline. We hypothesised that cigarette smoking would predict CUD, at both time points. We also investigated whether the effects of cannabis use on cannabis dependence were mediated by cigarette smoking.

Cigarette smoking at baseline was predictive of CUD at baseline when controlling for cannabis use variables in young people who smoke cannabis and tobacco. The most efficient model accounted for 30% of the variance in cannabis dependence which is considered to be a large effect size as *R* > 0.5 (Cohen, 1988). However, this seems no longer the case four years later, where only baseline CUD predicted follow-up CUD, accounting for almost 25% of the variance and replicating previous findings (Swift et al., 2000). When we investigated how cigarette smoking predicted concurrent CUD; we found that cigarette smoking (years of cigarette smoking and days per month cigarette smoking) mediated the relationship between cannabis use and cannabis dependence suggesting a role of tobacco use in the pathogenesis of CUD in cannabis and tobacco users. We also found these effects to be robust when controlling for depression and premorbid IQ (which were found to be comparable to normative values). Although causality cannot be assumed in this cross-sectional analysis, these results suggest that cigarette smoking may enhance the dependence-forming effects of cannabis. Alternatively, our results may suggest that CUD (as measured by the SDS) may capture some aspects of nicotine dependence in a subset of young people with CUD. As such, this research supplements previous epidemiological research that stresses the predictive ability of tobacco smoking in developing CUDs (Coffey et al., 2000; Prince van Leeuwen et al., 2014; von Sydow et al., 2002).

Our results, based in a naturalistic setting, parallel results from a recent controlled lab study that found cannabis users who smoke cigarettes are more likely to relapse in comparison to those who do not smoke cigarettes, perhaps as a result of this indirect pathway (de Dios et al., 2009; Haney et al., 2012). As such, reducing cannabis dependence might be facilitated by helping individuals quit cigarette smoking (Macleod et al., 2004). We were able to account for about 30% of the variance in CUD from four predictor variables. However, CUD is a complex disorder and causality cannot be determined from one factor. There are many other factors that can predict CUD that were beyond the scope of the current study but have interesting implications. For example, a recent study by van der Pol et al. (2013) found that current problems (such as living alone, coping motives for cannabis use and negative life events) were better predictors of cannabis dependence in young adults than cannabis exposure itself. As a result of this study, we included demographics and scores on the CTQ to our most efficient model, however these did not account for a significant proportion of variance to be included in the final model or in the mediation analysis. It is clear that CUD is a complex disorder that has many predictors and vulnerability factors that were not included in the model.

In the past, regular cigarette smoking would precede cannabis use (Kandel et al., 1992). This sequence in drug use seems to be tapering off, for example, around 1 in 5 young cannabis users have never smoked a cigarette (Suris et al., 2007). Interestingly, both cannabis and tobacco smoking were initiated 4.9 and 4.7 years previously, respectively, at the baseline visit, suggesting simultaneous age of onset in the current study. Therefore, these results do not speak to sequential use as on average the sample initiated both substances at the same time.

Stricter tobacco laws in some countries have altered perceptions such that cigarette smoking is considered a more risky behaviour than previously. In 2013, for the first time, tobacco smoking prevalence was estimated to be below 20% in the UK (Brown and West, 2014). In comparison, cannabis use has become normal and perceptions of regular cannabis use as a risky behaviour are at an all-time low (Johnston et al., 2013) with risk perception inversely related to prevalence of cannabis use (Kleber and Dupont, 2012). This may be due to the shifting landscape and debate over legalisation of both medical and recreational marijuana in states such as Colorado, California and Washington in the United States as well as countries such as Uruguay and the Netherlands (Volkow et al., 2014). As a result, whilst tobacco smoking decreases generally, it is possible that tobacco use will also increase indirectly over time due to increased cannabis use (Patton et al., 2005). Our findings are timely because they suggest tobacco may be involved in the pathogenesis of CUD, a possible risk factor of legalisation.

Our results may be a product of the common liability to the use of cannabis and tobacco including such risk factors like shared genetic and temperamental factors (Agrawal et al., 2008, 2010; Brook et al., 2010; Creemers et al., 2009). For example, recent research shows that nicotine dependence was associated stronger with lifetime CUD for females than males (Blanco et al., 2014). Moreover, Cooper and Haney (2014) have recently demonstrated that whilst subjective effects are equal across genders, females report more abuse related effects. Thus, an interesting analysis would be to investigate whether the mediators suggested in the present study, were stronger in females than males however, given that the sample was 71% male, this was not possible. Demographic variables were instead added to the most efficient model and we found that gender and age did not predict cannabis dependence after accounting for cannabis and tobacco use. Our results may also be a product of the common route of administration (Agrawal and Lynskey, 2009) where inhalation of one substance may sensitise an individual to the inhalation of another substance.

### 4.1 Strengths and limitations

This study has several strengths including a relatively large sample size of 298 young cannabis and tobacco users assessed in their own homes. Moreover, we used continuous variables to index both cannabis and tobacco smoking making it possible to assess the relationship between drug use variables at varying levels of severity (Ramo et al., 2013). This study also suffers from several limitations. First, within our exploratory follow-up sample we had a modest response from 65 participants. This may have reduced the power to detect a possible true effect of baseline cannabis use on future dependence (for example, surprisingly, days of cannabis use per month at baseline were not associated with cannabis dependence at follow up) and therefore these exploratory follow-up results should be interpreted with caution until they can be replicated with a greater sample size. Moreover, we were unable to control for the simultaneous use of cannabis and tobacco (joints) as the route of administration and as a necessity our sample is limited those who only smoke cannabis and tobacco. These results should be interpreted within their self-reported context. Finally, the multiple mediation analysis was conducted on cross sectional data and therefore the existence and direction of causality cannot be discerned.

### 4.2 Conclusions

In light of the medicalisation and legalisation of marijuana, research on cannabis and tobacco use is essential. In a naturalistic study of cannabis and tobacco co-users, baseline cigarette smoking (frequency and years) predicts cannabis dependence concurrently when controlling for frequency of cannabis use; however this was no longer the case four years later. At baseline, cigarette smoking mediated the relationship between cannabis use and cannabis dependence, even when controlling for psychological and demographic correlates that might explain this relationship. This suggests that cigarette smoking enhances vulnerability to the harmful effects of cannabis.

## Role of funding source

Funding for this study was provided by the Medical Research Council. The MRC had no further role in study design; in the collection, analysis and interpretation of data; in the writing of the report; or in the decision to submit the paper for publication.

## Contributors

Authors H.V.C. and C.J.M. designed the study. Author C.H. managed literature searches and summaries of previous related work. Authors G.L., T.P.F., N.D.S. and G.G. undertook data collection. C.H., N.D.S., T.P.F., C.J.F., and R.K.D. undertook the statistical analysis, and authors C.H. and N.D.S. wrote the first draft of the manuscript. All authors contributed to and have approved the final manuscript.

## Conflict of interest

All authors declare that they have no conflicts of interest.

## Figures and Tables

**Table 1:** Means, standard deviations and correlation coefficients of the primary baseline study variables.

	SDS	Time to smoke 3.5 g	DAYS-CANNABIS	Years cannabis used	Cannabis last used (days)	DAYS-TOB	YEARS-TOB	Tobacco last used (days)	Cigarettes per day
SDS	1.00	−0.19**	0.50***	0.14*	−0.25***	0.30***	0.26***	0.00	0.20**
Time to smoke 3.5 g		1.00	−0.40***	−0.04	0.18**	−0.11	0.00	0.01	−0.08
DAYS-CANNABIS			1.00	0.13*	−0.46***	0.33***	0.18**	0.01	0.24***
Years cannabis used				1.00	−0.17**	0.13*	0.59***	0.09	0.16**
Cannabis last used (days)					1.00	−0.16	−0.07	−0.02	−0.10
DAYS-TOB						1.00	0.24***	−0.18**	0.44***
YEARS-TOB							1.00	−0.09	0.19**
Tobacco last used (days)								1.00	−0.09
Cigarettes per day									1.00

*M*	2.85	8.51	18.50	4.94	3.76	23.61	4.70	20.10	7.17
SD	2.73	15.00	10.68	2.34	6.05	10.01	2.53	111.85	5.86

* *p* < .05.

** *p* < .01.

*** *p* < .001.

**Table 2:** Predicting cannabis dependence from cannabis exposure variables only, tobacco exposure variables, and psychological correlates (confounders) to develop the most efficient model.

	*B*	BCa 95% CI (lower, upper)	*p*
Cannabis only			
*R*^2^ = 0.246, *p* < 0.001			
Constant	0.221	−0.65, 1.11	0.632
Time to smoke 3.5 g	0.001	−0.02, 0.23	0.929
**DAYS-CANNABIS**	**0.120**	**0.09, 0.15**	**<0.001**
years cannabis used	0.090	−0.03, 0.21	0.144
cannabis last used	−0.012	−0.06, 0.03	0.537
Cannabis + tobacco			
*R*^2^ = 0.285, *F* = 23.93, *p* < 0.001			
Constant	−0.400	−1.34, 0.51	0.415
Time to smoke 3.5 g	−0.002	−0.02, 0.18	0.866
**DAYS-CANNABIS**	**0.101**	**0.07, 0.13**	**<0.001**
Years cannabis used	−0.054	−0.18, 0.09	0.407
Cannabis last used	−0.020	−0.07, 0.03	0.321
**DAYS-TOB**	**0.031**	**0.00, 0.06**	**0.020**
**YEARS-TOB**	**0.197**	**0.06, 0.32**	**0.003**
Tobacco last used	0.001	0.00, 0.03	0.174
Cigarettes per day	0.010	−0.04, 0.07	0.728
Cannabis, tobacco + confounders			
*R*^2^ = 0.304, *F* = 12.52, *p* < 0.001			
Constant	0.560	−1.30, 2.30	0.552
Time to smoke 3.5 g	−0.001	−0.02, 0.02	0.934
**DAYS-CANNABIS**	**0.098**	**0.07, 0.13**	**<0.001**
Years cannabis used	−0.022	−0.15, 0.11	0.766
Cannabis last used	−0.023	−0.07, 0.03	0.363
DAYS-TOB	0.030	0.00, 0.06	0.057
**YEARS-TOB**	**0.175**	**0.04, 0.31**	**0.012**
Tobacco last used	0.001	−0.05, 0.05	0.488
Cigarettes per day	0.010	−0.07, 0.01	0.984
WTAR	−0.029	0.01, 0.40	0.160
**BDI**	**−0.046**	**0.00, 0.09**	**0.033**
Most efficient model			
*R*^2^ = 0.295, *F* = 30.72, *p* < 0.001			
Constant	−0.933	−1.62, −0.28	0.008
**DAYS-CANNABIS**	**0.107**	**0.08, 0.13**	**<0.001**
**DAYS-TOB**	**0.029**	**0.01, 0.05**	**0.010**
**YEARS-TOB**	**0.159**	**0.05, 0.27**	**0.006**
**BDI**	**0.050**	**0.01, 0.10**	**0.020**

**Table 3:** Means, standard deviations and correlation coefficients of the primary baseline study variables with follow up cannabis dependence (*n* = 65).

	Baseline					Follow up
	SDS	DAYS-CANNABIS	DAYS-TOB	YEARS-TOB	BDI	SDS
Baseline						
SDS	1.00	0.57***	0.42**	0.29*	0.12	0.37**
DAYS-CANNABIS		1.00	0.39**	0.29*	0.14	0.26*
DAYS-TOB			1.00	0.31*	0.14	0.23
YEARS-TOB				1.00	−0.07	0.09
BDI					1.00	0.37**
Follow-up SDS						1.00

*M*	2.80	19.25	23.53	5.25	6.51	1.40
SD	2.64	10.63	9.93	2.31	5.59	2.29

* *p* < .05.

** *p* < .01.

*** *p* < .001.

**Table 4:** Predicting cannabis dependence at follow up from variables that predicted baseline cannabis dependence i.e. the most efficient model and assessing whether they still account for the model when cannabis dependence at baseline is added as a factor.

	*B*	BCa 95% CI (lower, upper)	*p*
Most efficient model			
*R*^2^ = 0.139, *F* = 1.85, *p* < 0.135			
Constant	−0.708	−2.25, 0.59	0.409
DAYS-CANNABIS	0.039	−0.03, 0.10	0.164
DAYS-TOB	0.010	−0.04, −0.06	0.748
YEARS-TOB	0.042	−0.19, 0.28	0.741
BDI	0.137	−0.44, 0.32	0.265
Most efficient model accounting for baseline dependence			
*R*^2^ = 0.266, *F* = 3.264, *p* < 0.013			
Constant	−0.361	−1.88, 0.94	0.668
DAYS-CANNABIS	0.008	−0.06, 0.07	0.770
DAYS-TOB	−0.006	−0.51, 0.04	0.852
YEARS-TOB	0.014	−0.21, 0.25	0.910
BDI	0.138	−0.05, 0.32	0.294
**Baseline SDS**	**0.274**	**0.05, 0.53**	**0.023**
